# Multistep Optimization of β-Glucosidase Extraction from Germinated Soybeans (*Glycine max* L. Merril) and Recovery of Isoflavone Aglycones

**DOI:** 10.3390/foods7070110

**Published:** 2018-07-13

**Authors:** Luciane Yuri Yoshiara, Tiago Bervelieri Madeira, Adriano Costa de Camargo, Fereidoon Shahidi, Elza Iouko Ida

**Affiliations:** 1 Food Science Department, Londrina State University, Rod. Celso Garcia, KM 380, 86051-990 Londrina, PR, Brazil; lyoshiara@hotmail.com (L.Y.Y.); elida@uel.br (E.I.I.); 2 Chemistry Department, Londrina State University, Rod. Celso Garcia, KM 380, 86051-990 Londrina, PR, Brazil; madeiratb@gmail.com; 3 Department of Biochemistry, Memorial University of Newfoundland, St. John’s, NL A1B 3X9, Canada; fshahidi@mun.ca

**Keywords:** endogenous enzyme, phenolic compounds, ultra-high performance liquid chromatography, response surface methodology

## Abstract

Epicotyls from germinated soybeans (EGS) have great potential as sources of endogenous β-glucosidase. Furthermore, this enzyme may improve the conversion of isoflavones into their corresponding aglycones. β-Glucosidase may also increase the release of aglycones from the cell wall of the plant materials. Therefore, the aim of this work was to optimize both the extraction of β-glucosidase from EGS and to further examine its application in defatted soybean cotyledon to improve the recovery of aglycones, which were evaluated by ultra-high performance liquid chromatography (UHPLC). A multistep optimization was carried out and the effects of temperature and pH were investigated by applying a central composite design. The linear effect of pH and the quadratic effect of pH and temperature were significant for the extraction of β-glucosidase and recovery aglycones, respectively. Optimum extraction of β-glucosidase from EGS occurred at 30 °C and pH 5.0. Furthermore, the maximum recovery of aglycones (98.7%), which occurred at 35 °C and pH 7.0–7.6 during 144 h of germination, increased 8.5 times with respect to the lowest concentration. The higher bioaccessibility of aglycones when compared with their conjugated counterparts is well substantiated. Therefore, the data provided in this contribution may be useful for enhancing the benefits of soybean, their products, and/or their processing by-products.

## 1. Introduction

Isoflavones are recognized in human health due to several biological properties, whereas in plants these bioactive compounds also have antifungal properties, thus protecting the plants against plant pathogens [1]. Epidemiological studies have shown that isoflavones may help reduce the risk of some chronic diseases [2]. In humans, a higher incidence of some types of cancers (e.g., breast and colon cancer) and heart disease has been observed in Western populations that consume lower amounts of soy isoflavones compared with the Asian population [3]. According to Brandi [4], isoflavones may reduce bone loss, decrease the risk of development of osteoporosis, and attenuate the symptoms of menopause in women. Because of their structural and molecular weight similarity to the group of female hormones secreted by ovarian cells (estrogens), isoflavones are also known as phytoestrogens [5]. 

Soybean isoflavones can be found in 12 different forms: β-glycosidics, which have a glucose unit linked to the benzene ring (e.g., daidzin, genistin, and glycitin); β-glycosidic conjugated forms, acetylglycosidics (e.g., acetyldaidzin, acetylgenistin, and acetylglycitin), and malonylglycosidics (e.g., malonyldaidzin, malonylgenistin, and malonylglycitin); and the aglycone forms (e.g., daidzein, genistein, and glycitein), which are not linked to glucose [6]. However, the contribution of the conjugated forms in soybeans are higher compared with that of the aglycone counterparts [7]. Furthermore, soybeans are regarded as the only high-level edible source of isoflavones. The distribution and content of different isoflavones are influenced by multiple factors, such as genetic variety, growth location, and crop year [8]. Likewise, isoflavone contents may vary among different soybean seed components. In fact, only minor amounts of isoflavones are present in the seed coat whereas the hypocotyls contain high concentrations and the cotyledons have been reported to contain 80–90% of the total isoflavones in the seed [9]. However, despite all these variations, the potential health benefits of soybeans as sources of isoflavones are well substantiated.

Walsh et al. [10] conducted an in vitro experiment to screen the stability and bioaccessibility of isoflavones from soy bread. According to these authors, micellarization may be required for optimal bioaccessibility of isoflavones in the aglycone forms. Furthermore, they suggested that the bioavailability of isoflavones from foods containing fat and protein may exceed that from supplements due to enhanced bile secretion. Therefore, due to the high protein content in soybeans, soybean products may be better sources of bioaccessible isoflavones. Isoflavones in the aglycone form are absorbed faster than their conjugated counterparts; therefore, they may render higher biological activities than the latter ones [11,12]. 

β-glucosidase catalyzes the hydrolysis of conjugated isoflavones, thus generating their respective aglycones. During the hydration of soybeans, this enzyme hydrolyzes glycosidic isoflavones, which affords their respective aglycones [13]. β-Glucosidase (β-d-glucoside glucohydrolase, EC 3.2.1.21), which is commonly found in plants or as part of the metabolism of fungi and bacteria [14], catalyzes the hydrolysis of β-glycosidic di- and/or other glycoside conjugates and oligosaccharides from phenolic compounds, thus releasing both the sugar moiety and the aglycone. Because of this property, a high interest in applying β-glucosidase to increase the amount of aglycone isoflavones in soybean and soy products has been noted [15,16]. In intact plant tissues, β-glucosidases are stored in compartments separated from the substrate, thus playing an important role in the physiology of the plant. During germination, these enzymes act during the process of degradation and lignification of the cell walls of the plant material. Furthermore, they also act as plant growth regulators and during the activation of compounds related to the plant defense [17,18].

The activation of enzymes (e.g., β-glucosidase) occurs during germination [19]. Therefore, germinated soybean components may be a good source of endogenous β-glucosidase which can be extracted and further applied in soy products, thus improving the final products due to higher contents of highly bioaccessible phenolic bioactives [20]. In epicotyls from germinated soybeans (EGS), the specific activity of the crude β-glucosidase extract has been reported to be 72-fold higher than that of the cotyledon extract from ungerminated soybeans and 5.8-fold higher than that of the crude cotyledon extract from germinated soybeans under the same conditions. Therefore, epicotyls have been recommended as a potential industrial source of endogenous β-glucosidase for hydrolysis of conjugated isoflavones to obtain aglycones [21].

The extraction of endogenous β-glucosidases and the use of this enzyme for isoflavone conversion have not yet been well explored in comparison to those of enzymes of microbial origin. Furthermore, the central composite design (CCD) method is an important experimental design used in response surface methodology to construct a second-order model for the response variable without needing a complete three-level factorial experiment, thus reducing the number of experiments while providing trustworthy results. Response surface methodology (RSM) has been successfully used for developing, improving, and optimizing different processes, including the procurement and/or extraction of phenolic compounds [22,23,24]. Therefore, due to the importance of β-glucosidase, and the existing knowledge gap in the literature, the objective of this work was to use CCD to optimize both the extraction of β-glucosidase from EGS and to establish the best conditions to convert glycosidic isoflavones into their corresponding aglycones by treatment with extracts containing β-glucosidase. 

## 2. Materials and Methods 

### 2.1. Materials

Soybeans (cv. BRS 257) were developed by Embrapa Soybean (Londrina, PR, Brazil). Aglycones (daidzein, glicitein, and genistein) and acetylglucosides standards (daidzin, glycitin, genistin) were purchased from Sigma-Aldrich (Saint Louis, MO, USA). The remaining solvents and chemicals were of analytical or HPLC grade. 

### 2.2. Soybean Germination Process

A previous study was used to select the germination conditions [21]. In this process, 15 germination paper rolls with 50 seeds each were placed in a germination chamber (Marconi, MA 835, Brazil). The seeds were then subjected to a photoperiod of 10 h of light per day. The temperature was kept at 35 °C (±1 °C) and controlled relative humidity (100%) for 144 h [21]. The epicotyls were manually separated, freeze-dried (Christ, ALPHA 1-4 LD plus, Germany), ground (A11 Basic Mill, Ika, Brazil), and stored at −26 °C until analysis.

### 2.3. CCD-Based Optimization of Extraction of Active β-Glucosidase

The effect of temperature (*X*_1_ = 23, 25, 30, 35, and 37 °C) and pH (*X*_2_ = 3.6, 4.0, 5.0, 6.0, and 6.4) on the extraction of active β-glucosidase from EGS was evaluated by applying the CCD with 5 levels of variation in a total of 11 assays (Table 1). Therefore, the response function (*Y*) stems from the β-glucosidase activity. The extraction procedure was performed as described by Carrão-Panizzi and Bordingnon [25] using 0.1 mol L^−1^ sodium citrate buffer with 0.1 mol L^−1^ NaCl at various pH values (*X*_1_) and temperatures (*X*_2_), according to the experimental design. The extraction procedure (100 mg of EGS in 1.5 mL sodium citrate buffer) was conducted under agitation for 50 min. After centrifugation at 2500× *g* (Cientec, CT 600, Piracicaba, Brazil) for 15 min, the extract so obtained was used for determination of β-glucosidase activity. 

### 2.4. CCD-Based Optimization of β-Glucosidase-Assisted Conversion of Conjugated Isoflavones into Their Corresponding Aglycones

Extracts with the highest β-glucosidase activity were used in this step of the experiment. A CCD with 2 variables (*X*_3_ = temperature and *X*_4_ = pH) and 5 variation levels (Table 2) in 2 blocks was used. The first block consisted of a 2^2^ factorial design, a total of 7 assays (assays 1–7), and 2 variables with 3 variation levels (*X*_3_ = 20, 35, and 50 °C and *X*_4_ = pH 4.0, 5.5, and 7.0). The second block, with a total of 6 assays (assays 8–13) containing the axial points of the CCD (*X*_3_ = 13.9 and 56.2 °C and *X*_4_ = pH 3.39, and 7.61), was performed to verify the quadratic effects. Previous experiments were conducted (data not shown) and the maximum conversion was achieved at 14 h of hydrolysis. The assays were performed randomly with defatted soybean cotyledon flours in 3.0 mL buffer (1:10 *w*/*v*). According to each design, the pH values were adjusted with 0.1 M sodium phosphate and 0.1 M citric acid solutions. To each test tube, 5.0 units of β-glucosidase activity from EGS were added and shaken for 14 h at temperatures described in Table 2. The samples were freeze-dried and used for identification and quantification of isoflavones by ultra-high performance liquid chromatography (UHPLC). The response function stems from the percentage of aglycones (*W* = % aglycones) obtained in relation to the total isoflavone content present in each sample. 

### 2.5. Determination of β-Glucosidase Activity

The enzymatic activity was determined according to the method described in the literature [26] with slight modifications. Briefly, 0.4 mL of 16 mM *p*-nitrophenyl-beta-D-glucopyranoside and 0.1 M phosphate–citrate buffer (pH 5.0) were transferred to a test tube and placed in a water bath at 30 °C for 10 min. The sample (0.1 mL) was added and the test tubes were placed in a water bath at 30 °C for 30 min more. The reaction was terminated with the addition of 0.5 M sodium carbonate (0.5 mL). The concentration of *p*-nitrophenol released during the reaction was determined by reading the absorbance at 420 nm with a spectrophotometer (Biochrom Libra S22, Cambridge, England). For quantification, a *p*-nitrophenol (20–160 mM) calibration curve was prepared. One unit of enzyme activity (UA) was defined as the amount of β-glucosidase that releases 1 mM *p*-nitrophenol min^−1^. The results were expressed as units of β-glucosidase activity (UA mL^−1^) per milliliter of extract.

### 2.6. Extraction and Determination of Isoflavones by Ultra-High-Performance Liquid Chromatography (UHPLC) 

Defatted cotyledon soy flours subjected to enzymatic conversion were used for extraction. Water/ethanol/acetone (1:1:1 *v*/*v*/*v*) was used for the extraction of isoflavones. The samples (250 mg) were mixed with 6 mL of solvent and the extraction was performed under sonication at 60 °C for 10 min [27]. After centrifugation and filtration (Millex–LH filters; 0.20 µm) of the supernatant, the extracts (1.4 µL) were injected into the UHPLC (Acquity UPLC® System), with automatic system injection, oven with controlled temperature at 35 °C, and a diode array detector (Waters, Milford, MA, USA). A reversed-phase column, BEH C18 (Waters, 2.1 mm × 50 mm, 1.7 µm particles), was used. The binary mobile phase consisted of acidified water (glacial acetic acid, pH 3.0)—mobile phase A—and acetonitrile—mobile phase B. The elution gradient used was as follows: 0 min, 90% A and 10% B; 8 min, 0% A and 100% B. The initial condition was re-established at 9 min. The total run took 12 min and the flow rate was 0.70 mL min^−1^. The temperature was kept constant (35 °C) and a diode array detector (Waters) with a wavelength set at 260 nm was used. Isoflavones, namely daidzin, glycitin, genistin, daidzein, glicitein, and genistein, were identified and quantified by comparing their retention times and UV spectra with coded and authentic standards under the same conditions as the samples. The presence of acetyldaidzin, acetylgenistin, and acetylglycitin was also investigated with coded and authentic standards. However, they were not detected in any of the samples tested and/or treatments; this is common for raw soybeans [6]. According to the literature [28], malonylglucosides (malonyldaidzin, malonylglycitin, and malonylgenistin) were quantified based on the standard curves of the corresponding β-glycosidic isoflavones (daidzin, glycitin, and genistin, respectively) using the similarity of the extinction coefficients. Limits of detection and quantification for listed compounds ranged from 0.003 to 0.0239 and from 0.009 to 0.725 µg/mL, respectively. Regression coefficients of the plotted graphs had *R*^2^ ranging from 0.9988 to 0.9996.

### 2.7. Statistical Analysis

STATISTICA 8.0 software (StatSoft, Palo Alto, CA, USA) was used to determine the effects of independent variables, calculate the regression coefficient (*R*^2^), perform analysis of variance (ANOVA), and build the response surfaces at 5% significance. Data were adjusted to a second-order polynomial model (Equation (1)):

(1) $$y=\;\beta_{0}+\;\beta_{1}x_{1}+\;\beta_{2}x_{2}+\;\beta_{11}x_{1}^{2}+\;\beta_{22}x_{2}^{2}+\beta_{12}x_{1}x_{2}$$

where *y* is the response variable; *x*_1_ and *x*_2_ are the coded process variables; and *β*_0_, *β*_1_, *β*_2_, *β*_11_, *β*_22_, and *β*_12_ are the regression coefficients.

To evaluate and validate the mathematical models, a new assay was performed under the conditions (*X*_1_ = °C and *X*_2_ = pH) that yielded the extracts with higher activity of β-glucosidase from EGS and the highest conversion of conjugated isoflavones into their respective aglycones (*X*_3_ = °C and *X*_4_ = pH). The observed model was obtained under experimental conditions, and the calculated values (*ŷ* and *ŵ*) were determined using the proposed model. The model was validated and the observed responses were within the confidence interval of the model. 

## 3. Results and Discussion 

### 3.1. Optimization of β-Glucosidase Extraction from Germinated Soybean Epicotyls

The linear effect of the variable *X*_1_ (temperature) on the response function *y* (β-glucosidase activity) was not significant, whereas the linear effect of the variable *X*_2_ (pH) was significant. In contrast, the quadratic effects of the variables *X*_1_ and *X*_2_ were significant but the interaction between the variables *X*_1_ and *X*_2_ was not significant (Table 3). These results indicated that variable *X*_2_ (pH between 3.6 and 6.4) was essential to obtaining extracts with high β-glucosidase activity from epicotyls from germinated soybeans. 

The determination coefficient (*R*^2^) of 0.94 indicates that 94% of the experimental data fitted the model. The polynomial model (*Y*) representing the activity of β-glucosidase of extracts from EGS is described below:

*Y* = 11.39 + 0.18*x*_1_ − 1.29*x*_1_^2^ − 1.97*x*^2^ − 3.48*x*_2_^2^ + 0.12*x*_1_*x*_2_ (2)

where *x*_1_ and *x*_2_ are the coded variables representing the temperature and pH, respectively, for the optimum recovery of high-activity β-glucosidase extracts from EGS.

**Figure 1:**
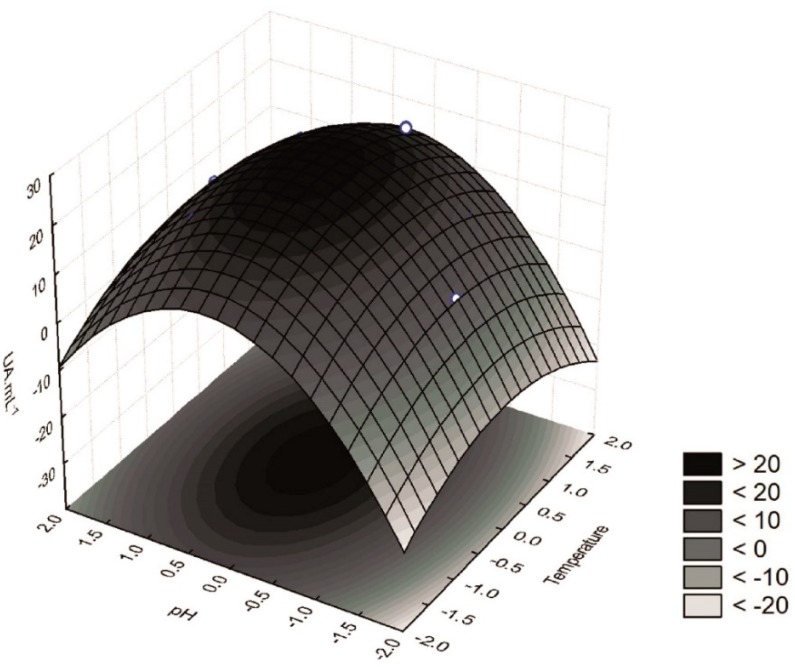
Surface response for the activity of β-glucosidase (UA mL^−1^) from epicotyls from germinated soybeans.

**Figure 2:**
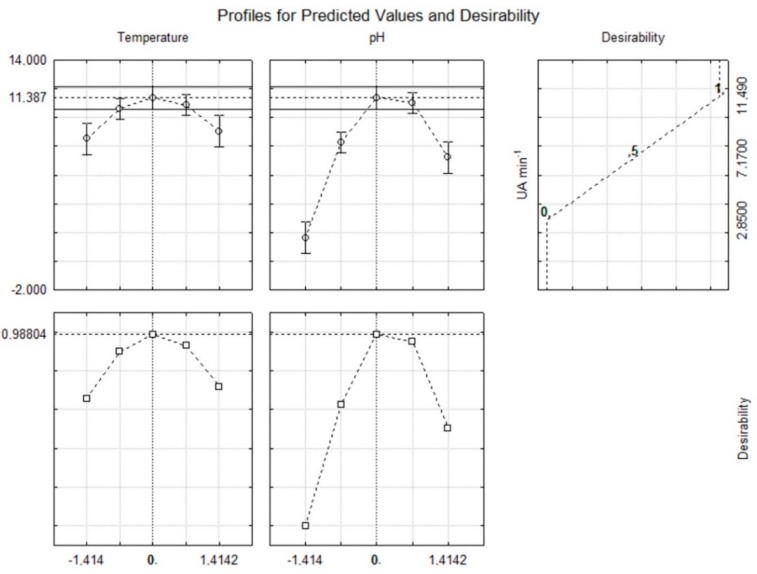
Profiles for predicted values and desirability for β-glucosidase activity of extracts from epicotyls from germinated soybeans.

According to the results in Table 4, extracts obtained at 30 or 35 °C and low pH values of 3.6 or 4.0 (assays 10 and 3, respectively) showed lower β-glucosidase activity. In contrast, higher β-glucosidase activity was found in extracts obtained at 23 °C and pH 5.0 (assay 8), 25 °C and pH 6.0 (assay 2), 30 °C and pH 6.4 (assay 11), and 37 °C and pH 5.0 (assay 9). Finally, the response surface (Figure 1) shows that the highest β-glucosidase activity was obtained when the extractions were performed at the central point, i.e., at 30 °C and pH 5.0 (assays 5, 6, and 7). Furthermore, extracts with high β-glucosidase activity could also be obtained at temperatures ranging from 27.5 to 33.5 °C and pH from 4.9 to 5.5. The conditions used as the central point were also in good agreement with the desirability parameter of the proposed model (Figure 2), thus supporting the procurement of extracts with a high β-glucosidase activity at 30 °C and pH 5.0.

Matsuura and Obata [26] extracted, partially purified, and characterized β-glucosidases from soybean cotyledons under different conditions including lower temperatures (between −10 and 5 °C). Although the extraction of β-glucosidase from cotyledons has been studied by these authors, to the best of our knowledge, the procurement of extracts with high β-glucosidase activity from germinated soybean epicotyls has not been reported in the literature. Furthermore, our proposed model proved to be advantageous to establish the best conditions to obtain extracts with high enzyme activity. In addition, working at 30 °C offers more operational advantages compared to the lower temperatures (between −10 and 5 °C) reported in the literature [26]. The proposed model was validated with an additional experiment under optimal conditions and the results (Figure 4) fell within the confidence interval of the estimated response, thus confirming the validity of the model.

### 3.2. Optimization of the Recovery of Aglycones Using β-Glucosidase from Germinated Soybean Epicotyls

According to variance analysis (ANOVA), the linear effect of the variable *X*_4_ (pH) and quadratic effects of the variables *X*_3_ (°C) and *X*_4_ (pH) were significant. However, the effects of the block, linear variable *X*_3_ (°C), and the interaction of the variables *X*_3_ (°C) and *X*_4_ (pH) on the response (*W* = % aglycones) were not significant (Table 5). Therefore, nonsignificant terms were excluded from the model which did not affect the *R*^2^. The coefficient of determination (*R*^2^) was 0.86, in other words, 86% of experimental data can be explained by the model. Therefore, Equation (3) is described as follows:

*W* = 81.938 + 22.328*x*_4_ − 11.231*x*_4_^2^ − 13.8562*x*_3_^2^ (3)

where *W* = % aglycones and *x*_3_ and *x*_4_ are the coded variables representing the temperature and pH, respectively.

The aglycone content of the soybean cotyledon flour (BRS 257) devoid of β-glucosidase addition was 2.9% and, regardless of the temperature (*X*_3_), reached values higher than 47% (Table 6) with greater pH (*X*_4_) values (0, +1, and +1.41). This suggests a greater pH influence on the response function (*W* = % of aglycones), thus supporting the screening of the main effects. 

**Figure 3:**
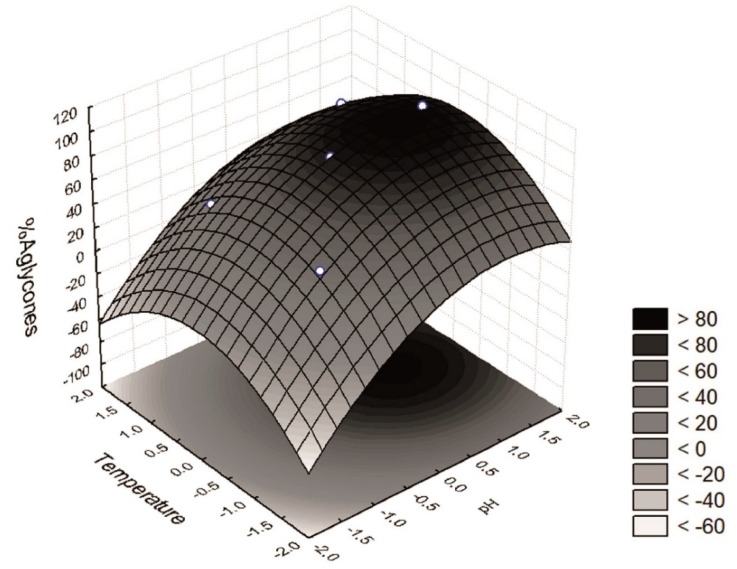
Surface response for the conversion of glycosidic isoflavones into their corresponding aglycones. High-aglycone defatted soybean cotyledon flours were produced by treatment with β-glucosidase from germinated soybean epicotyls.

**Figure 4:**
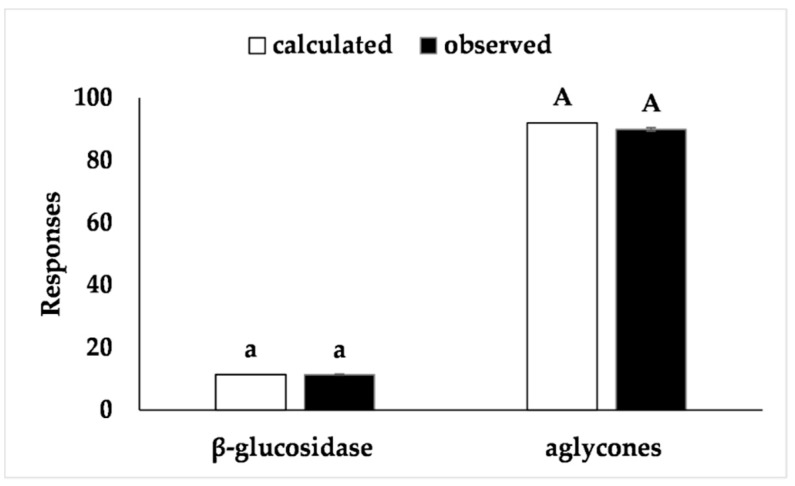
Validation of the models proposed for the procurement of extracts with β-glucosidase from epicotyls from germinated soybeans and high-aglycone defatted soybean cotyledon flours. Means with the same lower case (β-glucosidase) or capital (aglycones) letters: results fall within the confidence interval of the generated model.

The response surface (Figure 3) shows a region at which more than 80% of the isoflavones recovered were in the aglycone form, i.e., a pH range between +0.5 and +1.5 (6.25 < pH < 7.75) and a temperature between −0.5 and +0.5 (27.5 < T < 42.5). Furthermore, an optimum region at which the maximum percentage of isoflavones in the aglycone form was recovered could be determined. The desirability parameter indicates two optimal points of maximum (*W* = 98.7% aglycones), i.e., when *x*_3_ = 0 (35 °C) and *x*_4_ = +1 or +1.41 (pH = 7.00 and 7.61). One of these test points coincides with assay 9 (*Z* = 98.7% aglycones). These results are distinct from the optimum conditions of pH (between 5.2 and 6.0) and temperature (50 °C) for β-glucosidase activity from soybean cell tissue described by Hosel and Todenhagen [29] and by Matsuura and Obata [30] who reported optimal activity at pH 4.5 and a temperature of 45 °C for a soybean β-glucosidase. However, the proposed models were validated for both response functions which were within the confidence interval of the model (Figure 4).

A complete conversion of glycosidic isoflavones into their aglycones has been described by other authors [31]. However, their study was conducted with β-glucosidase from microbial origin (*Lactobacillus* (L.) *rhamnosus* CRL981). Furthermore, Song and Yu [32] applied β-glucosidase from *Thermotoga maritima* to recombinant soybean flour and obtained an almost complete conversion of all isoflavone glycosides. Endogenous β-glucosidases represent a more secure source of β-glucosidases than microbial sources. However, endogenous β-glucosidases have been poorly explored when compared with the microbial conversion of conjugated isoflavones into their aglycone forms. As mentioned before, in the present study, the maximum aglycone isoflavones obtained was 98.7% when endogenous β-glucosidase from EGS was used under the two conditions described above, therefore providing strong evidence on the potential use of EGS as a great source of endogenous β-glucosidase. 

Several pieces of evidence have demonstrated that different plant parts may present different phenolic compounds [33,34,35,36,37,38,39], therefore suggesting that the expression of genes associated with the production of some enzymes may be tissue specific. Likewise, not only the absorption of the phenolics is important but also their structure/activity [40,41,42]. Perera et al. [40] demonstrated that epigallocatechin gallate derivatives procured via lipophilization showed enhanced antioxidant properties compared with those of epigallocatechin gallate. Likewise, Oh and Shahidi [42] reported that several lipophilized resveratrol derivatives had better hydrogen peroxide scavenging activity than resveratrol. In addition, Oldoni et al. [41] demonstrated that minor changes such as the position of the hydroxyl group in flavonoids may be responsible for major changes in their final effects. According to their study, the concentration of procyanidin A2 necessary to scavenge 50% of the DPPH radical was 1.7-fold higher than that of procyanidin A1. Besides the antioxidant activity, which may be related to the inhibition of low-density lipoprotein cholesterol oxidation and DNA damage [43,44], phenolic compounds have been regarded as potential inhibitors of enzymes related to the absorption of carbohydrates and lipids [38,45]. In a bioactivity-guided isolation and purification study to identify α-glucosidase inhibitors, Sun et al. [46] suggested that C_1_-OH of the saccharide moiety in phenolic glycosides are necessary for a potent inhibition of intestinal α-glucosidases. According to Bustanji et al. [47], the inhibitory effect towards lipase activity was in the order of gallic acid > caffeic acid > chlorogenic acid > rosmarinic acid. A recent study [37] also demonstrated that whereas proanthocyanidin-rich extracts showed higher antioxidant activity, the extracts containing only phenolic acids showed higher antimicrobial effects. Finally, inflammation has been linked to several health issues, including those related to oxidative stress. A myriad of phenolic compounds have been reported to act as potential anti-inflammatory and antioxidant compounds [39,48,49,50] and the link between inflammatory responses and several diseases is well recognized. According to Lee et al. [51], fermented soymilk with greater contents of isoflavones in the aglycone form also showed higher antioxidant properties. The importance of natural product characterization in studies related to their bioactivity has long been discussed [52,53]. Therefore, by understanding the best conditions to produce β-glucosidase from EGS as well as its further application in the procurement of high-aglycone defatted soybean cotyledon flours, the present study contributes to both basic and applied science related to food bioactives and health. 

## 4. Conclusions

EGS was demonstrated to be of great potential as a source of endogenous β-glucosidase. Furthermore, two models were optimized, both for the extraction of β-glucosidase from EGS and for further application in defatted soybean cotyledon flour. Optimum extraction of β-glucosidase from EGS was procured at 30 °C and pH 5.0 whereas the maximum recovery of aglycones (98.7%) occurred at 35 °C and pH ranging from 7.0 to 7.6. The higher bioaccessibility of aglycones when compared with their conjugated counterparts has already been discussed by other authors [54,55]. Furthermore, the structure/activity has also been in the spotlight. Therefore, by reporting the best conditions to obtain a high-aglycone soybean feedstock, the present contribution may be useful for enhancing knowledge about the potential benefits of soybean products and/or processing by-products.

## Figures and Tables

**Table 1:** Independent variables and variation levels for the central composite design for optimization of the extraction of active β-glucosidase from epicotyls from geminated soybeans.

Independent Variables	Variation Levels				
	−1.41	−1	0	+1	+1.41
*X*_1_ = Temperature (°C)	23	25	30	35	37
*X*_2_ = pH	3.6	4.0	5.0	6.0	6.4

**Table 2:** Independent variables and variation levels for the central composite design for optimization of the conversion of conjugated isoflavones into their corresponding aglycones.

Independent Variables	Variation Levels				
	−1.41	−1	0	+1	+1.41
*X*_3_ = Temperature (°C)	13.9	20.0	35.0	50.0	56.2
*X*_4_ = pH	3.39	4.00	5.50	7.00	7.61

**Table 3:** Analysis of variance (ANOVA) for the β-glucosidase activity of extracts obtained from epicotyls from germinated soybeans.

Variation Source	SS	DF	MS	F Test	*p*	*R* ^2^
*X*_1_ (T) (linear)	0.558	1	0.558	0.659	0.428	0.94
*X*_1_ (T) (quadratic)	19.038	1	19.038	22.492	0.002	
*X*_2_ (pH) (linear)	62.340	1	62.340	73.650	0.000	
*X*_2_ (pH) (quadratic)	137.038	1	137.038	161.900	0.000	
Interaction *X*_1_*X*_2_	0.120	1	0.120	0.142	0.711	
Error	13.543	16	0.846			
Total	214.527	21				

SS = sum square. DF = degrees of freedom. MS = mean square. T = temperature in °C.

**Table 4:** Central composite design with two coded (*x*_1_ and *x*_2_) and decoded (*X*_1_ and *X*_2_) variables and the response function (*Y*) for the activity of β-glucosidase from epicotyls from germinated soybeans.

Assays	Coded Variables		Decoded Variable		Response Function (*Y*)
	*x*_1_	*x*_2_	T (°C) (*X*_1_)	pH (*X*_2_)	β-Glucosidase Activity (UA mL^−1^)
1	−1	−1	25.0	4.0	8.16
2	−1	1	25.0	6.0	17.7
3	1	−1	35.0	4.0	6.22
4	1	1	35.0	6.0	16.7
5	0	0	30.0	5.0	22.4
6	0	0	30.0	5.0	23.0
7	0	0	30.0	5.0	23.0
8	−1.41	0	23.0	5.0	16.5
9	1.41	0	37.0	5.0	20.7
10	0	−1.41	30.0	3.6	5.76
11	0	1.41	30.0	6.4	13.9

**Table 5:** Analysis of variance (ANOVA) for the conversion of conjugated isoflavones into their corresponding aglycones.

Variation Source	SS	DF	MS	F test	*p*	*R* ^2^
Block	22.050	1	22.050	0.17864	0.683680	0.86
(*X_4_*) pH (Linear)	4217.201	1	4217.201	34.16513	0.000385	
(*X_4_*) pH (Quadratic)	872.759	1	872.759	7.07055	0.028848	
(*X_3_*) T (Quadratic)	1328.402	1	1328.402	10.76189	0.011179	
Error	987.486	8	123.436			
Total	7231.600	12				

SS = sum square. DF = degrees of freedom. MS = mean square. T = temperature in °C.

**Table 6:** Central composite design with two coded (*x*_3_ and *x*_4_) and decoded (*X*_3_ and *X*_4_) variables and the response function (*W*) for the conversion of conjugated isoflavones into their corresponding aglycones.

Assays	Block	Coded Variables		Decoded Variables		Response Function (*W*)
		*x*_3_	*x*_4_	T (°C) (*X*_3_)	pH (*X*_4_)	% Aglycones *
1	1	−1	−1	20.0	4.00	47.5
2	1	+1	−1	50.0	4.00	44.6
3	1	−1	+1	20.0	7.00	68.6
4	1	+1	+1	50.0	7.00	84.0
5 (c)	1	0	0	35.0	5.50	88.6
6 (c)	1	0	0	35.0	5.50	79.0
7 (c)	1	0	0	35.0	5.50	88.6
8	2	0	−1.41	35.0	3.39	11.6
9	2	0	+1.41	35.0	7.61	98.7
10	2	−1.41	0	13.9	5.50	48.6
11	2	+1.41	0	56.2	5.50	47.1
12 (c)	2	0	0	35.0	5.50	85.0
13 (c)	2	0	0	35.0	5.50	88.3

* % aglycone isoflavones relative to total isoflavones extracted, determined by ultra-high-performance liquid chromatography (UHPLC). High-aglycone defatted soybean cotyledon flours were produced by treatment with β-glucosidase from germinated soybean epicotyls. “c” is central point.
